# 6-Aza-2-Thiothymine as an Alternative Matrix for Spatial Proteomics with MALDI-MSI

**DOI:** 10.3390/ijms252413678

**Published:** 2024-12-21

**Authors:** Vanna Denti, Nicole Monza, Greta Bindi, Natalia Shelly Porto, Vincenzo L’Imperio, Fabio Pagni, Isabella Piga, Andrew Smith

**Affiliations:** 1 Proteomics and Metabolomics Unit, Department of Medicine and Surgery, University of Milano-Bicocca, 20900 Monza, Italy; vanna.denti@unimib.it (V.D.); n.monza@campus.unimib.it (N.M.); g.bindi@campus.unimib.it (G.B.); n.porto@campus.unimib.it (N.S.P.); 2 Pathology Unit, Department of Medicine and Surgery, Fondazione IRCCS San Gerardo dei Tintori, University of Milano-Bicocca, 20900 Monza, Italy; vincenzo.limperio@unimib.it (V.L.); fabio.pagni@unimib.it (F.P.)

**Keywords:** 6-aza-2-thiothymine, MALDI-MSI, proteomics, spatial omics

## Abstract

Matrix Assisted Laser Desorption/Ionisation-Mass Spectrometry Imaging (MALDI-MSI) is a well-established spatial omic technique which enables the untargeted mapping of various classes of biomolecules, including tryptic peptides, directly on tissue. This method relies on the use of matrices for the ionisation and volatilisation of analytes, and α-Cyano-4-hydroxycinnamic acid (CHCA) represents the most widespread matrix for tryptic peptides analysis. However, CHCA also presents certain limitations that foster the quest for novel matrix compounds. 6-aza-2-thiothymine (ATT), traditionally used in MALDI mass spectrometry (MS) for oligonucleotides, small molecules and oxidised phospholipids, has not been thoroughly investigated as a potential matrix for tryptic peptide analysis in MALDI-MS or MALDI-MSI. Therefore, this study addresses this gap by evaluating the capability of ATT to ionise tryptic peptides from Bovine Serum Albumin (BSA) and map in situ-digested peptides from formalin-fixed paraffin-embedded (FFPE) tissue sections in these respective applications. Comparative analysis with CHCA demonstrated the complementary strengths of these matrices for detecting tryptic peptides, establishing ATT as a feasible alternative to CHCA in the MALDI-MSI field and paving the way for future advancements in spatial proteomics.

## 1. Introduction

Matrix Assisted Laser Desorption/Ionisation-Mass Spectrometry Imaging (MALDI-MSI) represents a well-established and reliable technique for the untargeted mapping of a wide array of biomolecules, including peptides, lipids, metabolites, oligonucleotides and xenobiotics, directly on tissue [1,2]. In the realm of this technique, there is a consistently high demand for novel matrix compounds that can enable the desorption and ionisation of analytes, enhancing signal-to-noise ratio whilst preserving spatial localisation within the intricate landscape [3,4] of tissue. The quest for novel MSI analytical methodologies is particularly relevant in the context of spatial proteomics, where detecting differential protein expression whilst mapping tryptic peptide distribution in situ can enable the study of molecular mechanisms underpinning disease [5] and provide deeper insights into the molecular landscape of pathological tissue. α-Cyano-4-hydroxycinnamic acid (CHCA) stands out as one of the most popular MALDI matrices for the analysis of tryptic peptides in MALDI-MSI experiments [6], representing the gold standard for spatial proteomic applications. Notwithstanding, this matrix presents some limitations, including the formation of matrix clusters ([aM + bAlkali − (b − 1)H]^+^) [7] within the lower peptide mass range (*m*/*z* 700–1200), which can hinder peptide detection and complicate data interpretation [8,9,10]. These clusters typically follow a general formula of [aM + bAlkali − (b − 1)H]+ (a = 1, 2, 3, …; b = 1, 2, 3, …) and are often accompanied by additional ions formed through the loss of water ([aM + bAlkali − (b − 1)H-H_2_O]^+^) or carbon dioxide ([aM + bAlkali − (b − 1)H-CO_2_]^+^), further complicating spectral analysis [7]. Moreover, CHCA has a limited capacity to volatilise analytes of higher molecular weight, reducing the amount of information available in the higher peptide mass range (*m*/*z* 1700–3000) [11]. Since this characteristic of CHCA is well known, solution additives and tissue washes with ammonium buffers have been explored, proving to decrease these clusters [8,12]. Nonetheless, in light of these weaknesses, alternative matrices should be considered for MALDI-MSI analysis. Among the array of well-established MALDI matrices, the pH-neutral 6-aza-2-thiothymine (ATT) matrix has traditionally found application in MALDI-MS, offering versatility by functioning in both positive and negative ionisation modes, while being characterised by minimal matrix peak cluster formation. Its adaptability extends to a diverse array of molecules, particularly oligonucleotides [13,14], small molecules [15], carbohydrates [16], glycosphingolipids [17] and oxidised phospholipids [18]. Moreover, the combination of ATT with additives such as pyridine has already been proven to enhance sensitivity and spot-to spot repeatability for oligonucleotides analyses [19]. Furthermore, few studies have highlighted its utility in the field of proteomics, including the analysis of intact or digested glycoproteins [20,21], as well the analysis of protein complexes [22]. In light of these findings, the primary goal of this study was to assess ATT’s capacity for ionizing tryptic peptides whilst considering their spatial distribution. In fact, despite the extensive use of ATT in sample preparation for traditional MALDI-MS analysis, thus far its application as the MALDI-MSI matrix was confined to the mapping of xenobiotics [15,23] and small molecules, such as single amino acids [15], using a 150 µm spatial resolution. Therefore, in this proof-of-concept study, we revisit the efficacy of ATT as a potential matrix for the analysis of tryptic peptides in MALDI-MS and MALDI-MSI. The study assesses the applicability of ATT for the peptide mass fingerprinting of Bovine Serum Albumin (BSA) and for the MALDI-MSI analysis of formalin-fixed paraffin-embedded (FFPE) clinical tissue, including thyroid cancer and clear cell renal cell carcinoma (ccRCC) samples; the results were compared with those obtained with CHCA, the gold standard matrix for the analysis of tryptic peptides with MALDI-MS. Our findings confirm the feasibility of ATT for tryptic peptide ionisations in the MALDI-MS and MSI realms, proposing it as a valid alternative to CHCA for the analysis of tryptic peptides and placing emphasis on their complementary strengths.

## 2. Results and Discussion

### 2.1. Peptide Mass Fingerprinting of BSA: Comparison of CHCA and ATT

**Figure 1:**
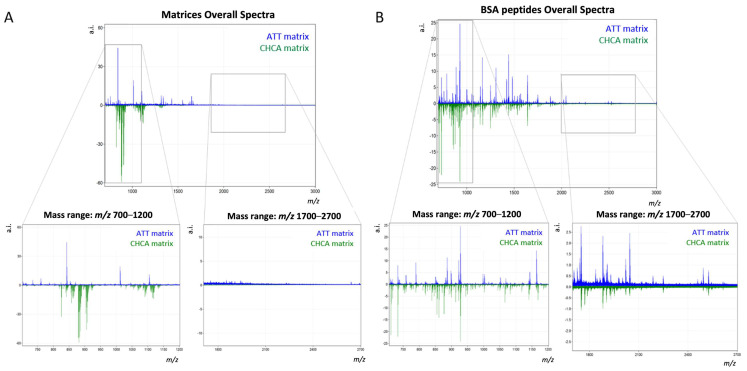
Comparative MALDI-MS analysis of the overall spectra of ATT (in blue) and CHCA (in green) (**A**) and tryptic peptides of BSA (**B**) analysed with the two matrices over the *m*/*z* range 700–3000. Differences in the spectra within the lower (*m*/*z* 700–1200) and the higher (*m*/*z* 1700–2700) mass ranges are highlighted.

In light of the previous applications of ATT for oligonucleotides [13,14], small molecules [15], and phospholipids [18] in MALDI-MS, its feasibility for the detection of tryptic peptides was consequently investigated. As a first step, spots of ATT and CHCA matrices were analysed with MALDI-MS to characterise their profiles. The average spectrum obtained from CHCA displayed characteristic clusters of matrix adducts in the lower *m*/*z* range (*m*/*z* 700–1200), whilst the higher portion of the mass range (*m*/*z* 1700–3000) revealed an absence of matrix peaks. Conversely, ATT yielded fewer and more sparse matrix peaks in the lower *m*/*z* range of the spectrum, and exhibited a few low-intensity peaks in the higher *m*/*z* range (Figure 1A). Notoriously, the presence of matrix clusters in the lower *m*/*z* range constitutes a pitfall of CHCA [24,25,26], as these could potentially interfere with the detection of coinciding tryptic peptides, thereby complicating the interpretation of spectra. Considering that ATT produced fewer and more scattered matrix peaks, and could therefore be considered a virtually “MALDI-silent” matrix [27] with fewer matrix related peaks exclusively in the low molecular weight spectral region (*m*/*z* 700–1200), these results highlight the potential of this matrix to overcome the drawbacks of CHCA in the lower portion of the mass range.

Subsequently, the effectiveness of ATT for the analysis of tryptic peptides was assessed by alternatively profiling digested bovine BSA with ATT and CHCA. While the obtained spectra presented notably different profiles, this analysis demonstrated the capability of ATT to effectively ionise tryptic peptides (Figure 1B). When acquired peptides were matched with theoretical masses obtained through in silico digestion of BSA, a comparable number of matches was assessed with ATT and CHCA. However, ATT enabled an increased number of peptide matches in the higher *m*/*z* range (*m*/*z* 1700–3000) and a greater BSA overall sequence coverage (47%) compared to CHCA (39%) to be observed (Table 1).

MALDI-MS peptide mass fingerprinting holds great potential for routine diagnostics, such as the analysis of neonatal dried blood spots for the early detection of genetic disorders [28], or within the healthcare setting [29] for the identification of microbes, including bacteria and fungi. Therefore, technical improvements that result in a higher protein sequence coverage represent a valid way to drive forward the use of MALDI-MS within the clinical setting.

Overall, ATT proved its efficacy in tryptic peptide desorption, ionisation and volatilisation, highlighting a comparable performance in respect to CHCA, which represents the gold standard matrix for MALDI-MS analysis of peptides. Notwithstanding, the reduced number of matrix clusters within the lower *m*/*z* range and the higher peptide sequence coverage obtained with ATT indicate this matrix as a promising alternative to CHCA for MALDI-MS peptide analysis.

### 2.2. Characterisation of ATT Matrix for MALDI-MSI Tryptic Peptides Analysis

**Figure 2:**
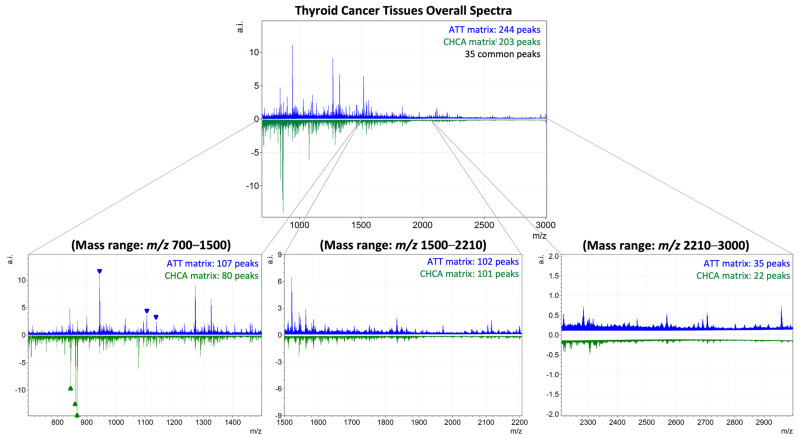
Comparative MALDI-MSI overall spectra of thyroid cancer tissues analysed with ATT (in blue) and CHCA (in green) matrices over the *m*/*z* 700–3000. The spectra are divided into low (*m*/*z* 700–1500), intermediate (*m*/*z* 1500–2210) and high (*m*/*z* 2210-3000) mass ranges, with the number of peaks identified in each range. Arrows indicate matrix adducts.

**Figure 3:**
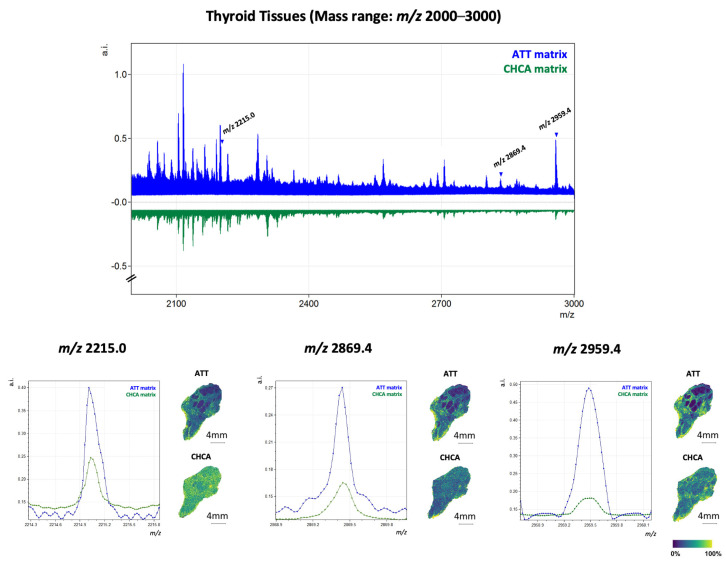
MALDI-MSI spectra of thyroid tissues in the high mass range (*m*/*z* 2000–3000) analysed with ATT (in blue) and CHCA (in green) matrices. Arrows indicate three peaks (*m*/*z:* 2215.0.0, 2869.4 and 2959.4) with a high mass-to-charge ratio. The absolute intensities (a.i.) and the spatial localization of these peaks are shown in the tissues under each analysed condition.

After assessing the capability of ATT to ionise tryptic peptides in MALDI-MS, the matrix was consequently evaluated, as a proof of concept, on on-tissue-digested FFPE thyroid cancer samples for MSI applications. The preliminary results obtained through MALDI-MS were confirmed with MSI, as ATT could enable a satisfying tryptic peptide signals yield, even when compared to CHCA (Figure 2). Specifically, the analysis of FFPE thyroid tissue using ATT revealed a higher number of peaks in the average spectrum (244 peaks) compared to those observed with the conventional gold standard matrix (203 peaks). As preliminarily assessed in MALDI-MS, this tendency was particularly pronounced in the high mass range (*m*/*z* 2210-3000), where the number of peaks identified with ATT was approximately double that of CHCA (35 and 22, respectively). To further elucidate this observation, three peaks within the high mass range (*m*/*z* 2000–3000) were selected for detailed analysis, specifically *m*/*z* 2215.0 [30], *m*/*z* 2959.4 [30], and *m*/*z* 2869.4 [30], as shown in Figure 3. The absolute intensities (a.i.) of these peaks, along with their spatial localisation, are presented for comparison. Notably, the peak intensities in the spectrum obtained with ATT were consistently higher compared to those detected with CHCA. As displayed in the corresponding MALDI-MSI images, the enhanced signal-to-noise ratio (S/N) observed with ATT also enabled a more specific tissue distribution to be detected. Additionally, when performing tissue segmentation with bisecting k-means, the cluster trees obtained on tissue sections analysed with ATT and CHCA reveal that ATT enables a more efficient clustering of pixels based on spectral similarity, with less branching structures compared to the cluster tree obtained with CHCA (Supplementary Figure S1 and Table S1).

In this scenario, ATT represents another valuable matrix of choice for the spatial proteomics MALDI-MSI analysis of FFPE tissues, with our results highlighting a similar number of tryptic peptide features detected with respect to CHCA. Moreover, the enhanced number and S/N of features detected in the higher *m*/*z* range, which also facilitates more defined spatial localisations, altogether demonstrates the potential of ATT to overcome the limitations traditionally associated with CHCA [8,9,10,11]. In this context, ATT could be a promising matrix to be utilised in the field of extracellular matrix (ECM) protein characterisation. ECM alterations and ECM remodelling are mechanisms underlying several types of cancer [31,32,33], but also neurodegenerative disorders [34], vascular [35] diseases and more; considering that larger collagen tryptic peptides generally exhibit high *m*/*z* values [36], ATT could be a helpful tool to unveil the mechanisms at the base of the interplay between ECM and diseases due to its capability to ionise and volatilise even high-molecular-weight peptides [37].

### 2.3. Limitations of ATT Matrix in MALDI-MSI Analysis

**Figure 4:**
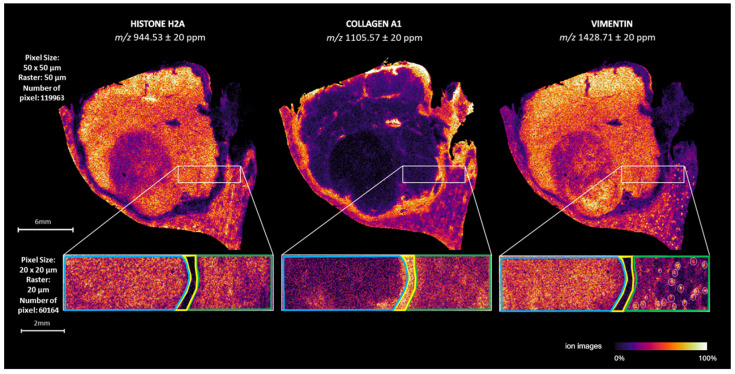
timsTOF fleXTM MALDI-MSI analyses of ccRCC tissues analysed with ATT matrix. Spatial localization of histone H2A (*m*/*z* 944.53), collagen alpha-1 (I) chain precursor (*m*/*z* 1105.57), and vimentin (*m*/*z* 1428.71) are shown in the whole tissue (raster 50 μm) and in the rectangle portion of tissue (raster: 20 μm).

The analyses conducted using ATT have demonstrated promising advantages when compared to the analyses that traditionally employed CHCA. However, according to practical observations, ATT seemed to undergo a certain degree of sublimation. Indeed, it is known that MALDI matrices can display high sublimation rates in (ultra) high vacuums, and this vacuum instability can interfere with the quality of the analysis over time [27,38]. Therefore, more extensive studies were performed to assess the gradual sublimation of ATT. Specifically, in the first instance, a 1-h MALDI-MSI acquisition was conducted on a defined, rectangular, sub-region of an FFPE ccRCC tissue sample using a raster setting of 20 µm (Figure 4; rectangular insets). Subsequently, 30 min after this first acquisition was concluded, a 2.36-h analysis was performed on the entire FFPE ccRCC tissue section with a raster setting of 50 µm. This second analysis commenced 1.5 h after matrix deposition, totalling 4 h of elapsed time. Three peaks—whose identity is well known in the field of MALDI-MSI — histone H2A (*m*/*z* 944.53) [30,39], Collagen alpha-1 (I) chain precursor (*m*/*z* 1105.57) [30,40], and vimentin (*m*/*z* 1428.71) [30,39], were examined to assess a possible loss in signal intensity, as highlighted in Figure 4. The intensity of these peaks across the entire tissue sample remained unaltered for the whole time of analysis, allowing us to obtain MALDI-MSI images of satisfying quality across the whole measurement region. For instance, 4 h after the matrix spray, it was still possible to discern glomeruli within renal tissue, even at a lower spatial resolution. Altogether, these results suggest that no matrix degradation occurs in less than 5 h following matrix spraying. To further inquire about the time required for matrix degradation to be observed, another spatial proteomics MALDI-MSI analysis, which lasted approximately 7.5 h, was performed on FFPE ccRCC tissues to assess possible matrix degradation in a long-term analysis. Results demonstrated that matrix degradation did not significantly impact the sensitivity of the analysis over time, with no signal loss detected (Supplementary Figure S2). The distribution of two matrix peaks was assessed as it was previously, indicating a satisfying distribution of the signals even in the absence of Total Ion Count (TIC) normalisation. As a final experiment, a non-digested tissue was placed on an ITO-coated slide, and small square portions of tissue were analysed at intervals of one hour for a total period of 5 h to assess exclusively the intensity of ATT peaks on tissue without considering tryptic peptides. The intensity of two matrix peaks was examined, and interestingly, a slight decrease of signal could indeed be detected hour by hour (Supplementary Figure S3). On the whole, these results indicate that ATT is a non-vacuum-stable matrix, but crystal stability is dependent on the presence of co-crystallised analytes; indeed, co-crystallised molecules could decrease the high volatility of ATT crystals, possibly lengthening their stability for longer-term analyses [3,41]. Altogether, these observations suggest that, while matrix degradation can be a concern in prolonged MALDI-MSI experiments, its effect on non-matrix peaks is minimal for at least 7.5 h after spraying, and it does not interfere with the accurate visualisation of molecular distributions within the tissue. In this context, the consistency of the results without normalisation (Supplementary Figure S2) underscores the stability of the signal, further indicating that matrix degradation has a limited influence on the reliability of molecular mapping of analytes. These findings have significant implications for the widespread application of MALDI-MSI in FFPE tissues, ensuring that the distribution of biologically relevant molecules can be accurately visualised, even over extended periods of analysis. However, in light of these findings, ATT may not be suitable for all applications, such as in tissue microarray (TMA) analysis, where consistency and reproducibility of signal intensity across multiple tissue samples are essential over a long period of time.

To conclude, this study evaluated the effectiveness of ATT for the analysis of tryptic peptides in MALDI-MS and MALDI-MSI applications. This matrix demonstrated significant utility in clinical biological samples investigation by enhancing ionisation efficiency in higher *m*/*z* ranges and minimising interference from matrix peaks. Overall, ATT could represent a promising alternative to the conventionally used CHCA, offering comparable performance whilst also partially compensating for its limitations. However, its low vacuum stability decreases its suitability for long-term analytical studies in vacuum MALDI platforms. Further optimization of the ATT matrix composition (i.e., use of different additives) and different matrix applications could enhance its stability. In different prospects, ATT could yield valuable insights to improve the mass spectral quality and enhance sensitivity in other omics applications, including lipidomics in MALDI-MSI, especially in light of previous results obtained in the analysis of oxidised lipids with MALDI-MS [18]. Nonetheless, further characterization of the physicochemical properties of ATT (e.g., crystal structure, ionization efficiency) are necessary to further understand its capabilities, improve the crystal dimension, and compensate for the current limitations.

## 3. Materials and Methods

### 3.1. Chemical and Reagents

High-performance liquid chromatography (HPLC)-grade toluene, HPLC-grade ethanol, HPLC-grade water, HPLC-grade acetonitrile (ACN) and HPLC-grade methanol (MeOH) were obtained from Honeywell SC, Seelze, Germany. Liquid chromatography-mass spectrometry (LC-MS) grade acetonitrile was obtained from LiChrosolv. Trifluoroacetic acid (TFA), guanidinium chloride (GUA), diammonium hydrogen citrate (DAHC) and trypsin from porcine pancreas were purchased from Sigma-Aldrich, Buchs, Switzerland. CHCA matrix and PepMix I were purchased from Bruker Daltonics, Bremen, Germany. ATT matrix was obtained from ChemCruz, Saint Cruz, CA, USA.

### 3.2. Sample Preparation for MALDI-MS

For MALDI-MS analysis, bovine serum albumin (BSA) was digested with trypsin from porcine pancreas (Sigma-Aldrich, Buchs, Switzerland). Subsequently, 1 μL of digested BSA (1:20 dilution in water) and 1 μL of CHCA matrix (10 mg/mL, 70:30 ACN:H_2_O, 1% TFA) or ATT matrix (10 mg/mL, 70:30 MeOH:H_2_O, 2 mM GUA, 20 mM DAHC) were spotted on an MTP 384 Target Plate Ground Steel (Bruker Daltonics, Bremen, Germany) with the dried droplet approach. Three replicates for each type of matrix were spotted. To test matrix degradation over time, several replicates of a mixture of BSA and CHCA or ATT (1:1) were spotted onto the target and analysed at intervals of 1 h (1–5 h).

### 3.3. Sample Preparation for MALDI-MSI

For MALDI-MSI analysis, FFPE thyroid carcinoma and ccRCC tissue samples were sectioned at a 5 µm thickness and placed onto conductive indium tin oxide (ITO) slides (Bruker Daltonics, Bremen, Germany). ITO slides were kept at room temperature and in the dark until the day of the study. Slides were processed according to our previously published method [42], which involves paraffin pre-melting at 65 °C for 1 h in an oven, dewaxing with toluene washes (3 × 5 min), tissue rehydration with decreasing concentrations of ethanol (EtOH:H_2_O 100:0, 70:30, 0:100), and finally, an acidic antigen retrieval (10 mM citric acid buffer, pH 5.95) in a water bath at 95 °C for 45 min. For tryptic digestion, an iMatrixSpray sprayer (Tardo Gmbh, Subingen, Switzerland) was used to apply homogeneous layers of trypsin (20 ng/μL). Tissues were then incubated overnight (for approximately 18 h) in a humidity chamber at 40 °C. After tryptic digestion, CHCA or ATT matrices were applied using an HTX TM-Sprayer (HTX Technologies, LLC, Chapel Hill, NC, USA). For both matrices, spraying parameters were set as follows: temperature 75 °C; number of passes 4 (for CHCA) and 3 (for ATT); flow rate 0.12 mL/min; velocity 1200 mm/min; track spacing 3 mm; and pressure 10 psi.

### 3.4. MALDI-MS and MALDI-MSI Analysis

A RapifleX MALDI Tissuetyper™ mass spectrometer (Bruker Daltonics, Bremen, Germany) equipped with a Smartbeam 3D laser operating at 5 kHz frequency was used to acquire both MALDI-MS mass spectra profiles of BSA and for the MALDI-MSI analysis of FFPE tissues.

Representative MALDI-MS mass spectra were acquired in reflectron positive mode in the mass range *m*/*z* 700–3000 using the random walk parameter of the sample carrier. For MALDI-MS spectra of trypsin-digested BSA spots, baseline subtraction (TopHat algorithm) and smoothing (Savitzky-Golay algorithm) were performed using FlexAnalysis software v.3.4 (Bruker Daltonics, Bremen, Germany). In order to evaluate the peptide coverage of BSA with both CHCA and ATT matrices, an in-silico digestion of BSA was performed using the open-source software mMass v.5.5 (http://www.mmass.org) [43]. Trypsin was set as the digestive enzyme with no fixed modifications, and methionine oxidation was selected as variable modification. The in silico-digested BSA peptides were subsequently matched with the ones presented in the MALDI-MS spectra of BSA, both obtained from the analysis with either ATT or CHCA.

MALDI-MSI images were obtained for each FFPE thyroid and ccRCC tissue sample using a raster width of 50 μm and a beam scan configuration of 46 × 46 μm. The MALDI-MSI analysis was performed in reflectron positive mode in the *m*/*z* 700–3000 range. For the external calibration, a mixture of standard peptides (PepMix I, Bruker Daltonics, Bremen, Germany) with a mass range of *m*/*z* 750–3150 was put directly onto the glass slide.

A timsTOF fleXTM (Bruker Daltonics, Bremen, Germany) was used to perform the MALDI-MSI analyses of two FFPE ccRCC tissue sections using either a raster width of 20 μm or 50 μm. A beam scan configuration of 16 × 16 μm or 46 × 46 μm was set, and 60,164 and 119,963 spots were acquired, respectively. The MALDI-MSI analysis was performed in reflectron positive mode in the *m*/*z* 700–3000 range with PepMix I as external calibration. The instrument parameters for the acquisition method were set up using the FlexControl v.4.0 (Bruker Daltonics, Bremen, Germany) and the timsControl v.5.1 (Bruker Daltonics, Bremen, Germany) for Rapiflex and TimsTOF fleXTM MALDI-MSI analyses, respectively. MALDI-MSI analysis visualisation was performed using the FlexImaging v.5.0 (Bruker Daltonics, Bremen, Germany).

### 3.5. MALDI-MSI Data Analysis

The data files containing the individual spectra of each entire measurement region were imported into SCiLS Lab 2024 Pro software (http://scils.de/Bruker Daltonics, Bremen, Germany), and preprocessing was carried out by performing baseline subtraction (TopHat algorithm), normalisation (Total Ion Current algorithm) and weak spatial denoising. The Bisecting K-Means algorithm was then used to perform hierarchical cluster analysis on pixels based on spectral similarity. The average (avg) spectra, representative of the whole measurement regions, were generated in order to display the differences in the protein profiles.

Average mass spectra were exported from SciLS Lab in txt format and imported into the open-source software mMass v.5.5 (http://www.mmass.org) for mass spectra visualisation.

## 4. Conclusions

In conclusion, in this proof-of-concept study, we assessed the efficacy of 6-aza-2-thiothymine as a suitable matrix for the analysis of tryptic peptides in MALDI-MS and MALDI-MSI applications. Specifically, ATT displayed comparable performance to CHCA, while also revealing complementary characteristics. On one hand, ATT could more efficiently ionise tryptic peptides of higher molecular weight, allowing for better BSA sequence coverage in MALDI-MS and more specific analyte distributions in MALDI-MSI in respect to CHCA. Moreover, this matrix presented less matrix peak clusters, making the interpretation of the lower tryptic peptide *m*/*z* range less challenging and compensating for CHCA’s proclivity for matrix adduct formation. In future work, the promising potential of spatially resolved MS/MS approaches, particularly with iprm-PASEF, could further advance this technology and help address limitations in direct MS/MS annotation of tissue proteins [44,45]. Additional efforts should focus on optimizing matrix deposition parameters specifically for ATT to ensure compatibility with the increased number of laser shots required for this method., Hence, while ATT may enhance spectral and molecular image clarity, its application should be evaluated based on the type of investigation and on the duration of the analysis. By optimizing the composition, crystal size, and stability of ATT, and investigating its ionization mechanisms, its utility in MALDI-MSI could be significantly expanded. While ATT complements the strengths and weaknesses of CHCA, its application should be tailored to the specific demands and duration of each investigation.

## Figures and Tables

**Table 1:** Number of matches obtained by confronting molecular signals from MALDI-MS and in silico digested.

		N° Matches				Average n° Matches	Sequence Coverage	Average Sequence Coverage
		*m*/*z* Range A	*m*/*z* Range B	*m*/*z* Range C	Total			
CHCA	Rep_1	22	5	0	27	32	32%	39%
	Rep_2	24	9	0	33		40%	
	Rep_3	25	9	1	35		44%	
ATT	Rep_1	18	10	4	32	34	45%	47%
	Rep_2	21	12	3	36		47%	
	Rep_3	18	11	5	34		49%	

## Data Availability

The original contributions presented in this study are included in the article/Supplementary Material. Further inquiries can be directed to the corresponding authors.

## Supplementary Materials

The following supporting information can be downloaded at: https://www.mdpi.com/article/10.3390/ijms252413678/s1.
