# Supplementary material for: 6-Aza-2-Thiothymine as an Alternative Matrix for Spatial Proteomics with MALDI-MSI
Source: Int J Mol Sci. 2024 Dec 21;25(24):13678. doi: 10.3390/ijms252413678 (PMC11678892; doi:10.3390/ijms252413678)
Supplement: Supplementary file 1 [file ijms-25-13678-s001.zip › ijms-3326013-supplementary.pdf]

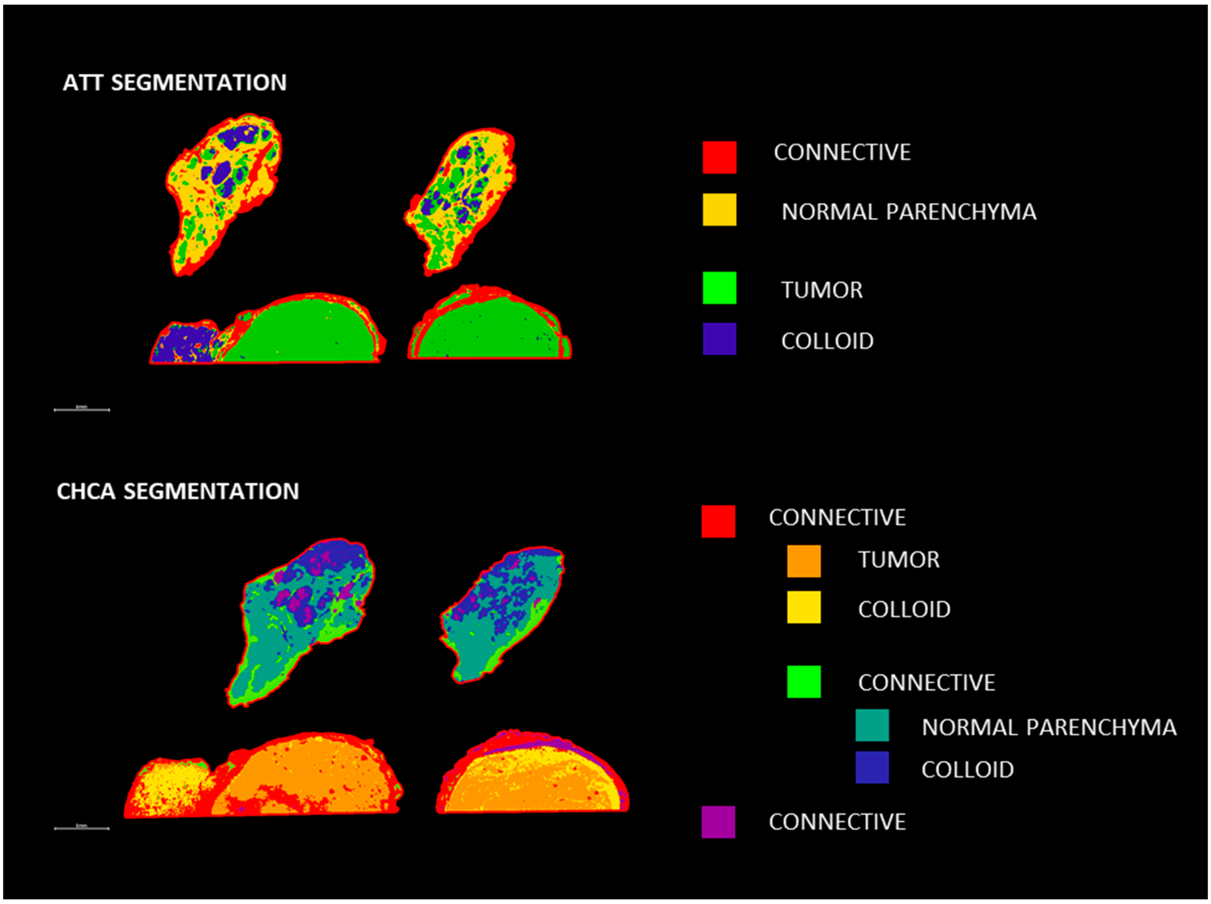

Figure S1. Bisecting K-Means segmentation cluster tree of the thyroid tissues analysed with ATT and CHCA, based on the proteomic data, allowed the distinction of different regions throughout the tissues.

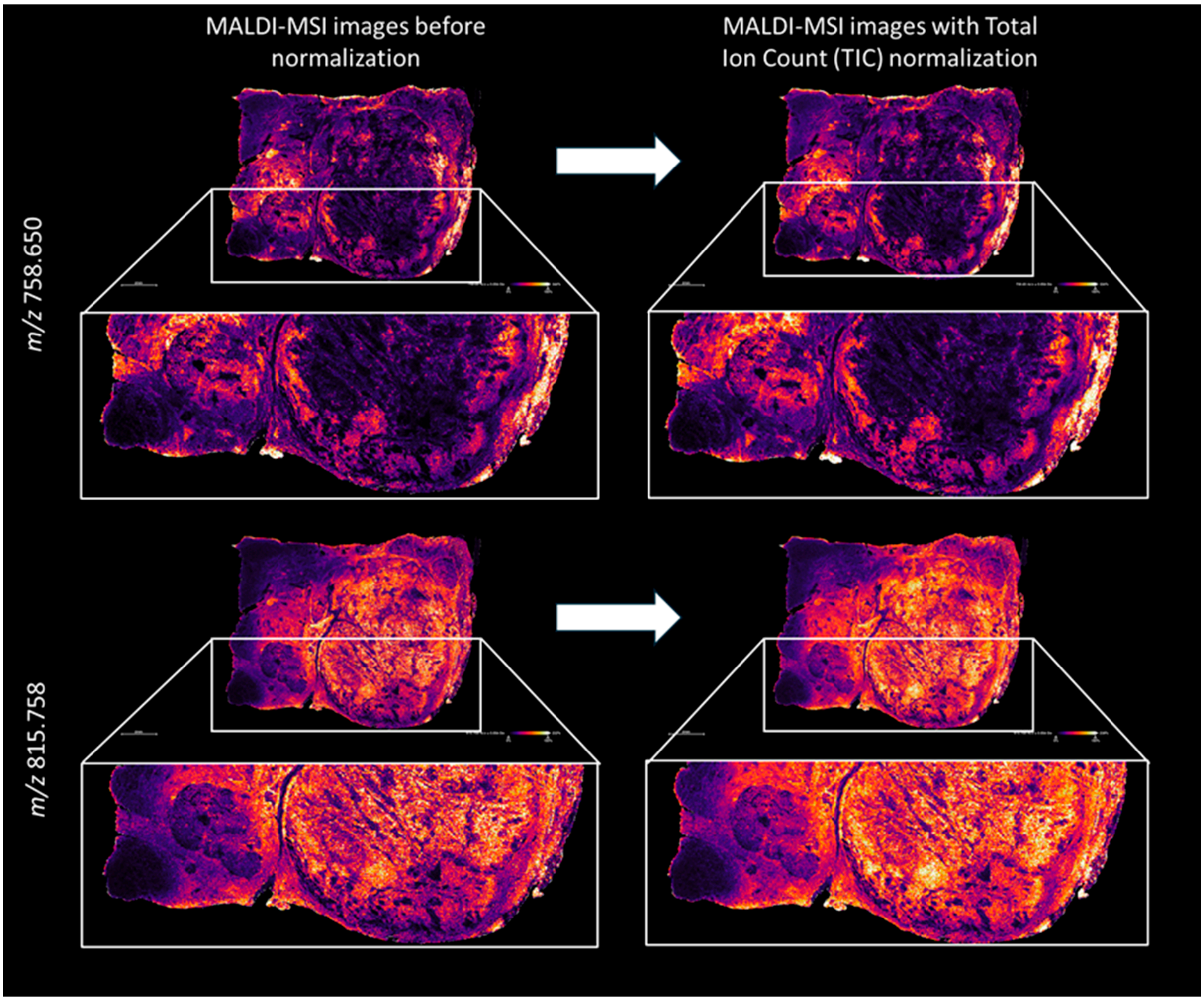

Figure S2. MALDI-MSI images of FFPE ccRCC tissue analysed with ATT matrix using RapifleX MALDI Tissuetyper mass spectrometer before (left panel) and after (right panel) Total Ion Count normalisation. Time of analysis ~ 7.5h.

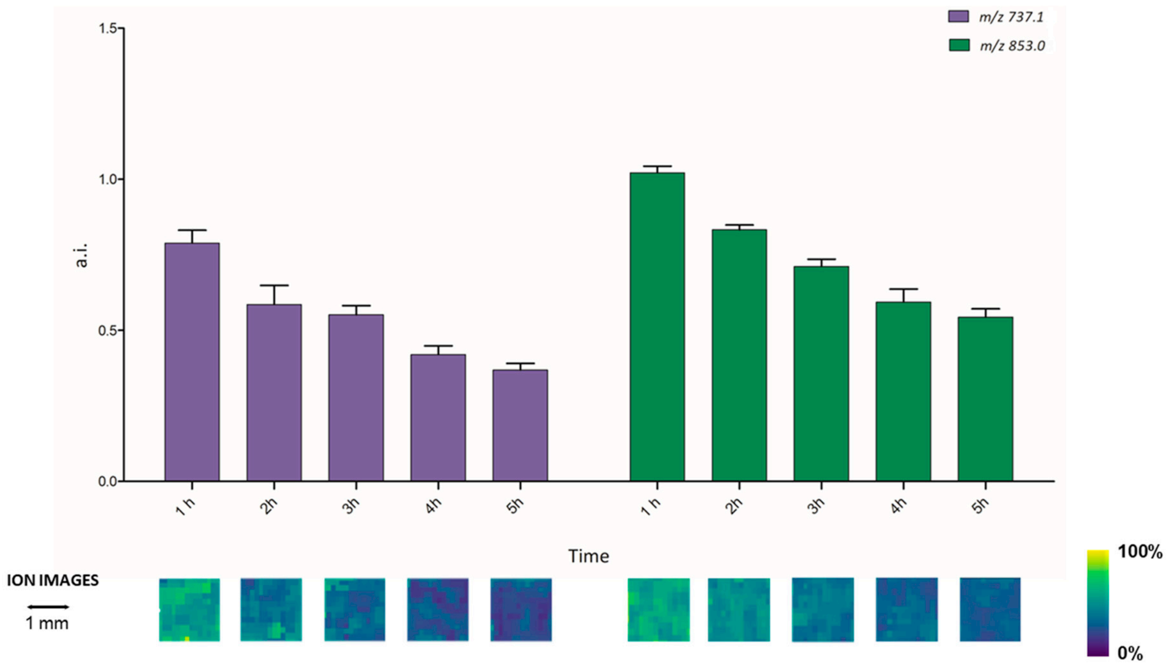

Figure S3. On the top, graphical representation of ATT sublimation – considering two ATT matrix peaks m/z 737.1 and 853.0 – on tissues up to 5 hours after matrix deposition. Time and absolute intensity (a.i) are presented in the X and Y axes respectively. On the bottom, ion images of the tissues section corresponding to the time of the analyses are shown.

Observed *m/z* (MALDI-MSI)	Expected *m/z*	Protein ID	Sequence	Δppm	ΔDa	
944.53	944.53	H2A1_HUMAN	r.AGLQFPVGR.v	2	0.00	*
1105.57	1105.58	CO1A1_HUMAN	r.VQGPPGPAGPR.g(Hydroxylated)	-9	-0.01	**
1428.71	1428.71	VIME_HUMAN	r.SLYASSPGGVYATR.s	-1	0.00	*
2215.00	2215.07	ACTB_HUMAN / ACTG_HUMAN	k.DLYANTVLSGGTTMYPGIADR.m	-32	-0.07	*
2869.43	2869.41	CO1A1_HUMAN	r.GLTGPIGPPGPAGAPGDKGESGPSGPAGPTGAR.g (Hydroxylated)	7	0.02	**
2959.43	2959.41	CO2A1_HUMAN	r.GPPGAAGAPGPQGFQGPAGEPGEPGQTGPAGAR.g (Hydroxylated)	8	0.02	**
2959.40	2959.66	RRBP1_HUMAN	R.APAVAVAPTPVQPPIIVAPVATVPAMPQEK.L	-87	-0.26	*

* identity confirmed with internal libraries nanoLC-MS/MS and or in situ MS/MS

** confirmed with literature data [1]

Table S1.

References

1. Groseclose MR, Massion PP, Chaurand P, Caprioli RM. High-throughput proteomic analysis of formalin-fixed paraffin-embedded tissue microarrays using MALDI imaging mass spectrometry. Proteomics. 2008;8: 3715–3724.
